# Editorial: Healthcare technologies and space: therapeutic built environment as a health technology and technologies for improved healthcare settings

**DOI:** 10.3389/fmedt.2025.1726059

**Published:** 2025-11-19

**Authors:** Evangelia Chrysikou, Fernando Loizides, Marianna Obrist, James Barlow, Paul Barach

**Affiliations:** 1 The Bartlett School of Sustainable Construction, University College London, London, United Kingdom; 2 Data Science Academy, Cardiff University, Cardiff, United Kingdom; 3 Department of Computer Science, University College London, London, United Kingdom; 4 Department of Economics and Public Policy, Imperial College Business School, London, United Kingdom; 5 College of Population Health, Thomas Jefferson University, Philadelphia, PA, United States; 6 Sheps Health Services Research Center, University of North Carolina, Chapel Hill, NC, United States; 7 Department of Surgery, Imperial College, London, United Kingdom

**Keywords:** healthcare technologies, built environment, therapeutic space, human factors, physical space, digital technologies

Healthcare built environments (BEs) are more than just structures for healthcare provision. For some, they are safe havens; for others, they are the locus of dynamic civic and financial activity; and for still others, they might be stressful places that might provide fragmented or even unsafe care (1, 2). These mixed messages have created interest in obtaining a greater understanding of the relationships between the quality of care and BE. Moreover, the discussion about human-technology interaction in the context of healthcare neglects the space of this interaction and overlooks the existential role of human factors engineering.

Human factors engineering is the application of knowledge about human behaviour, abilities, limitations to the design of BEs including the mechanical and software-driven user interfaces, systems, tasks, user documentation, and user training to enhance and demonstrate their safe and effective use (3). Healthcare workers perform under human factor constraints in complex, technology-infused, rapidly changing, time-constrained, and stressful work environments where effective performance demands expert knowledge, appropriate problem-solving strategies and fine motor skills (4).

Examining the healthcare BE or healthcare technologies in a systematic approach can shed light on the disconnects causing harm and undermining the potential of BE to deliver exceptional outcomes. The papers of this Special Issue are grouped under three, interrelated themes guided by the World Health Organisation's definition of health technology: “the application of organised knowledge and skills in the form of devices, medicines, vaccines, procedures, and systems developed to solve a health problem and improve quality of lives (5).”

The first theme collates contributions on the applications of organised knowledge and skills to solve a health problem. There is a shift towards hybrid care models, with online care becoming a growing tool for delivering and supporting at-home care. This generates a frequent need for infrastructure' retrofitting.

Scialpi and Declercq, focus on the definition of adaptability, using a hospital pilot to test the Reversible Building design protocol, and explore to what extent adaptability models are effective in orienting the design of healthcare buildings. The need for an architectural and systemic form of organised knowledge to address the complex healthcare structures is showcased.

Tan and Mills’ review explores challenges and opportunities in emergency department planning by integrating AI and multi-sensor research. Key challenges include data quality, space limits, and staff training. The study highlights the dynamic interrelations between healthcare technology and spatial design, suggesting that architecture can guide and become an integral part of healthcare technology to improve decision-making and efficiency.

Pilosof et al. redefines hospital infrastructure and virtual systems by examining how adaptable systems operate in Israel's hospital-at-home program for Internal Medicine patients. The authors explore the implications of hybrid service delivery for future BE designs and virtual technology platforms. The findings reveal a novel design approach, identifying challenges and synergies in enabling flexibility between physical spaces and remote technologies.

Smits et al. built and tested two prototypes of video consultation areas in an academic hospital in the Netherlands. They identified the end user's needs via co-design, BE, ergonomics requirements and technological needs for optimal usage. The study used an architectural case of hybrid care in practice, highlighting how an existing space can be successfully transformed into a hybrid care environment to best serve the many end-user's needs.

Keeping the momentum toward future-oriented, emerging applications, Yang explores how Mixed Reality Technology (MXR) can enhance learning and post-pandemic resilience in healthcare built environments. Drawing on a literature review and stakeholder interviews, it highlights synergies between architecture and technology, showing how MXR's capabilities can improve resource efficiency and support innovation in the healthcare sector.

Garcia-Sterling et al. position effective wayfinding as a vital health technology in healthcare settings, addressing diverse user needs. It emphasizes intuitive, efficient, and empathetic systems that accommodate neurodiversity, physical impairments, sensory loss, and life transitions like ageing. The authors introduce a user-centred digital wayfinding framework, reframing navigation in healthcare as both a functional and therapeutic experience that actively engages and supports patients and visitors.

Finally, Chrysikou et al. returning the narrative to digital transformation of people and to the effects on their wellbeing, explores the Digital Technologies (DTs) available to healthcare architects working within the NHS Estate in England or on NHS-related projects, and their impacts on wellbeing. As DTs become vital for design and communication, they can provide solutions to challenges in a profession with low job satisfaction and high turnover. The study highlights how digital tools can boost productivity, improve design quality, and enhance wellbeing, engagement, and resilience among healthcare architects.

The second theme focuses on how health technologies and the BE can improve the quality of life, promoting patient recovery and inclusion.

Tekin and Gutierrez apply a biophilic design concept as a structured framework, establishing the theoretical foundation for therapeutic environments. Focusing on UK non-clinical healthcare settings for cancer patients, the authors present a tailored conceptual framework. They offer spatial recommendations ranging from patient and staff perspectives, demonstrating how biophilic principles can enhance wellbeing across diverse therapeutic environments.

Wang and Tzortzi offer guidelines for implementing healing gardens as preventive medical tools in general hospitals. Using a hospital case study, the authors demonstrate how environmental design theory translates into practice. Highlighting the overlooked value of hospital outdoor spaces—often used as parking—as healing environments, the research provides a practical, cost-effective framework for garden design, especially relevant during and after public health crises like epidemics.

Yuan et al., endeavour to quantify the qualitative evidence, by evaluating hospital-affiliated green spaces' roles in patient recovery across three tertiary hospitals in China. The authors provide evidence for incorporating indirect wellness strategies into hospital design. Key design elements identified include hierarchical vegetation, road patterns, vibrant colors, accessibility features, and rehabilitation facilities. The research underscores the importance of built environment features in healthcare settings to enhance healing and support patient wellbeing.

Moving further to therapeutic components of the built environment, Palityka et al. explore art accessibility in healthcare for visually impaired people, often excluded from therapeutic benefits. While art aids wayfinding, reduces anxiety, and supports healing, most healthcare art programs remain inaccessible. The authors propose co-design strategies including involving visually impaired individuals in planning, integrating accessibility from the start, and fostering collaboration among artists, healthcare providers, and designers to create inclusive, engaging, and equitable art in clinical settings.

Rehn-Groenendijk et al., study how aesthetic design features influence health-related mental concepts and behaviour change. Two lecterns with different designs were tested in university and clinical orthopaedic rehab settings as primes. The research highlights the crucial role of design interventions in therapeutic environments and shows how an iterative, evidence-based approach can create effective design primes, offering valuable insights for promoting health behaviours through built environment strategies in future research.

Tracada and Allacci, extend the therapeutic design principles to everyday environments, proposing a broader definition of active, healthy living for all ages by evaluating shared building spaces. The authors developed an interdisciplinary Ratings Tool to Assess Inclusive Design for Self-Directed Healthy Behaviours, integrating urban planning, biophilia, active living, and social engagement. The tool supports creating inclusive common spaces that promote physical, social, and psychological wellbeing.

Minetou et al., closing this theme on a human note, present the design of spatial objects with narrative attributes to support the wellbeing of people living with dementia (PLWD). The authors explore how built environments affect dementia care quality. Three textile-based, technology-embedded prototypes were co-designed with PLWD to provide easily implementable tools in long-term care, activating stimuli sequences that engage and support residents effectively.

The third theme of this special issue expands the concept of applying health technology in alternative perspectives for improvements in life.

Phaisawang et al. examines the critical role of high-quality healthcare services and advanced technologies in ensuring the sector's economic sustainability. Focusing on health technology developers, the authors highlight their essential interdisciplinary skills—clinical knowledge, technical expertise, user-centered design, and business acumen— as key drivers of healthcare transformation. The findings emphasize the need for diverse learning and work competencies to build a sustainable, technology-driven healthcare ecosystem.

Gizachew et al. explore the intersections of wellbeing and technology by examining computer vision syndrome (CVS) among telecom employees in Ethiopia. Affecting over 70% of global computer users, CVS is influenced by environmental (lighting), work-related (screen brightness, viewing distance), and personal factors. The study assesses the determinants' impacts on CVS symptoms and offers practical recommendations to reduce its effects, emphasizing the need for healthier, tech-integrated work environments.

The studies collected in this Special Issue demonstrate that health technology extends well beyond traditional devices and procedures, positioning physical space as an active and integral component of care delivery. Through hybrid models, biophilic design, adaptive architecture, inclusive artistic interventions, and environments that promote social interactions and psychosocial wellbeing, the BE emerges as a form of health technology in its own right. The contributions firmly show that healthcare technologies and the BE are inseparable in the planning and delivery of modern health care. As cognitive demands and complexity increase, especially in digitally mediated care, new understandings of human–machine interactions are urgently needed. The science of human factors offers powerful tools to guide the design of spaces that support communication, coordination, and team integration—improving situational awareness, reducing errors, and enhancing provider wellbeing. From wayfinding systems and textile-based therapeutic objects to design frameworks for hybrid care environments, this collection shows how physical and digital systems have co-evolved. By positioning therapeutic environments as active participants in care, this Special Issue expands the definition of health technologies to include architecture, human factors, and the experiential and emotional qualities of place. Together, the contributions call for novel interdisciplinary, human-centred approaches to healthcare design—offering a more resilient, inclusive, and systemic vision for the future of care.
